# Application of Magnesium Modified Corn Biochar for Phosphorus Removal and Recovery from Swine Wastewater

**DOI:** 10.3390/ijerph110909217

**Published:** 2014-09-05

**Authors:** Ci Fang, Tao Zhang, Ping Li, Rong-feng Jiang, Ying-cai Wang

**Affiliations:** Key Laboratory of Plant-Soil Interactions of Ministry of Education, College of Resources and Environmental Sciences, China Agricultural University, Beijing 100193, China; E-Mails: fangci2012@163.com (C.F.); lipinglina@126.com (P.L.); rfjiang@cau.edu.cn (R.J.); yingcaiwangcau@gmail.com (Y.W.)

**Keywords:** phosphorus, magnesium modified corn biochar, swine wastewater, nutrient recovery

## Abstract

The recycling of lost phosphorus (P) is important in sustainable development. In line with this objective, biochar adsorption is a promising method of P recovery. Therefore, our study investigates the efficiency and selectivity of magnesium modified corn biochar (Mg/biochar) in relation to P adsorption. It also examines the available P derived from postsorption Mg/biochar. Mg/biochar is rich in magnesium nanoparticles and organic functional groups, and it can adsorb 90% of the equilibrium amount of P within 30 min. The Mg/biochar P adsorption process is mainly controlled by chemical action. The maximum P adsorption amount of Mg/biochar is 239 mg/g. The Langmuir-Freundlich model fits the P adsorption isotherm best. Thermodynamics calculation shows ∆H > 0, ∆G < 0, ∆S > 0, and it demonstrates the P adsorption process is an endothermic, spontaneous, and increasingly disordered. The optimal pH is 9. The amounts of P adsorbed by Mg/B300, Mg/B450, and Mg/B600 from swine wastewater are lower than that adsorbed from synthetic P wastewater by 6.6%, 4.8%, and 4.2%, respectively. Mg/biochar is more resistant to pH and to the influence of coexisting ions than biochar. Finally, postsorption Mg/biochar can release P persistently. The P release equilibrium concentrations are ordered as follows: Mg/B600 > Mg/B450 > Mg/B300. The postsorption Mg/B300, Mg/B450, and Mg/B600 can release 3.3%, 3.9%, and 4.4% of the total adsorbed P, respectively, per interval time.

## 1. Introduction

Intensive pig culture is currently an important part of the modern agricultural economy [1]. However, intensive pig culture plants usually generate a great deal of swine wastewater, which contain high concentrations of phosphorus (P) [2]. If this wastewater is not treated reasonably, it can facilitate mosquito breeding and the spread of bacteria in rural areas, especially if it is exposed to air. As a result, swine wastewater poses a major threat to public health. Environmentally, it does not only lead to the pollution of water eutrophication, but also wastes non-renewable resources [3]. Therefore, the P resource can be recycled from swine wastewater through innovative technology. 

Many such technologies have been developed for various applications, including biological P uptake, chemical precipitation, electrolysis, and adsorption [2]. Biological P uptake utilizes polyphosphate-accumulating organisms to capture P in their cells. However, this method is limited by the lack of carbon sources and the difficulty of culturing pure bacteria [4]. Chemical precipitation has been hindered by its high chemical consumption, low recovery efficiency, and excessive production of chemical sludge [5]. Electrolysis is restricted by the little wastewater treatment capacity and the need to replace electrodes frequently [6,7]. However, recovering P from wastewater using adsorbents is advantageous over the other methods because of its high efficiency and low consumption [2]. 

Biochar is a new pollution adsorbent that has received a great deal of attention recently. Many researchers have utilized biochar adsorption for organic contaminants, such as sulfamethoxazole [8], phenanthrene [9], and triazine pesticides [10], and heavy metals, such as lead [11], cadmium [12], and mercury [9]. 

Thus, biochar adsorption can be used to recover P from wastewater for nutrient recycling. However, the poor efficiency and selectivity of P adsorption hampers the application of typical biochar. Previous research derived low P recovery ratios of only 73% [13] and 37% [14]. Moreover, the coexisting ions in wastewater, including Cl^−^, NO_3_^−^, and HCO_3_^−^, can reduce the recovery ratio of selective P adsorption by 4%, 11%, and 41%, respectively [13]. Previous studies demonstrated cation modified function adsorbents, such as lanthanum/aluminum-modified zeolite adsorbent [15] and Fe-treated artificial zeolite [16], can enhance the efficiency and selectivity of P recovery. Cation-modified biochar has been developed recently. Chen* et al.* [17] loaded FeCl_2_ and FeCl_3_ on orange peel biochar to adsorb 1.24 mg/g of P. Zhang* et al.* [18] loaded AlOOH on cottonwood biochar and adsorbed 135 mg/g of P. 

The P from postsorption biochar is recycled as fertilizer to maximize nutrient resources in nutrient cycling. Hale* et al.* [14] reported that postsorption biochar could release 1.48 mg/g of reactive P, which was higher than the optimal P content required for plant growth (45–50 mg P/kg). However, the reuse of P as fertilizer may be affected by some of the cations that modify the biochar. Johnston and Richards [19] reported that the P in iron phosphates was hardly released and was unavailable for plant growth. Rittmann* et al.* [20] pointed out that aluminum was toxic to many plants and hardly released P as well. Therefore, the ease of P release and conduciveness to plant growth are important factors in the selection of cations for further biochar modification. 

Magnesium, involved in the chlorophyll formation of plants, is considered as a suitable cation for P recovery [13,21]. Thus, magnesium modified corn biochar (Mg/biochar) is prepared in our study. It is then compared with biochar, in terms of characteristics, P adsorption efficiency, selectivity in swine wastewater, and the available P of postsorption Mg/biochar. 

## 2. Experimental Section 

### 2.1. Materials 

Biochar: Ground corn was dipped in deionized water (DI) with a mass-to-volume ratio of 1:3 for 2 h, dried at 110 °C, and then pyrolyzed respectively at 300 °C, 450 °C, and 600 °C in nitrogen gas (limited oxygen) for 3 h (denoted as B300, B450, and B600, respectively). The pyrolyzed sample was cleaned by DI, dried at 60 °C, sieved through 0.1 mm–0.2 mm mesh, and sealed in a container before use. 

Mg/biochar: Ground corn was dipped in MgCl_2_ solution with a mass-to-volume ratio of 1:3 for 2 h, dried at 110 °C, and then pyrolyzed respectively at 300 °C, 450 °C, and 600 °C in nitrogen gas (limited oxygen) for 3 h (denoted as Mg/B300, Mg/B450, and Mg/B600, respectively). The pyrolyzed sample was cleaned by DI, dried at 60 °C, sieved through 0.1 mm–0.2 mm mesh, and sealed in a container before use. 

Swine wastewater: Raw swine wastewater was collected from a pig plant near Beijing. This wastewater was centrifuged, filtered, and stored in the refrigerator before use. The parameters of the swine wastewater are shown in Table 1. 

**Table 1:** The parameter of swine wastewater.

Items		Value
pH		7.8
COD	(mg·L^−1^)	11,850
TN	(mg·L^−1^)	397
NH_4_^+^-N	(mg·L^−1^)	365
PO_4_^3−^-P	(mg·L^−1^)	84
SS	(mg·L^−1^)	482
Ca^2+ ^	(mg·L^−1^)	23
Mg^2+ ^	(mg·L^−1^)	17
K^+ ^	(mg·L^−1^)	281
Na^+ ^	(mg·L^−1^)	58
Cl^− ^	(mg·L^−1^)	84
SO_4_^2− ^	(mg·L^−1^)	81

### 2.2. Methods

Kinetics adsorption: 0.2 g of either biochar or Mg/biochar was mixed with 20 mL swine wastewater, and then shaken at 200 rpm in 303 ± 0.5 K. The supernatant was collected after a certain time interval. 

Adsorption isotherm: 0.2 g of either biochar or Mg/biochar was mixed with 20 mL P solution (swine wastewater mixed with NaH_2_PO_4_ to obtain the initial P concentration of 84 mg P/L–2600 mg P/L), and shaken at 200 rpm for 12 h at 288 ± 0.5 K, 303 ± 0.5 K, and 318 ± 0.5 K. 

pH influence: 0.1 g of either biochar or Mg/biochar was mixed with 20 mL swine wastewater at an experimental pH range of 6–10. The mixture was then shaken at 200 rpm for 12 h at 303 ± 0.5 K. 

Influence of coexisting ions: 0.1 g of either biochar or Mg/biochar was mixed with 20 mL swine and synthetic P wastewater (P concentration 84 mg P/L) at a given experimental pH level of 9. The mixture was shaken at 200 rpm for 12 h at 303 ± 0.5 K. 

Continuous extraction: 0.1 g of either postsorption biochar or postsorption Mg/biochar (0.1 g of either biochar or Mg/biochar mixed with 2000 mL swine wastewater at 303 ± 0.5 K for 12 h) was combined with 120 mL DI containing 2% citric acid (citric acid DI), and shaken at 200 rpm at 303 ± 0.5 K. 

Interval extraction: after continuous extraction, 0.1 g of either postsorption biochar or postsorption Mg/biochar was mixed with 120 mL of freshly replaced citric acid DI, and shaken at 200 rpm at 303 ± 0.5 K. The supernatant was replaced with fresh extraction solution every 24 h. This interval extraction process was reiterated six times. 

### 2.3. Analysis

Biochar and Mg/biochar were analyzed by CHN element analyzer (vario EL, Hanau, Germany), Brunauer, Emmett, and Teller Surface Area Analyzer (BET, ASAP 2020, Atlanta, Georgia, USA), Fourier Transform Infrared Spectrometer (FTIR, Magna-IR 750, Washington, Maryland, USA), Transmission Electron Microscope (TEM, JEM-2100F, Tokyo, Japan). 

The concentrations of PO_4_^3−^-P were measured according to standard methods (APHA, 2012, 4500-PC. vanadomolybdophosphoric acid colorimetric method). All the experiments were repeated 3 times and the average values were calculated. 

## 3. Results and Discussion

### 3.1. Characterization Analysis

The characterization analysis demonstrated that an increase in the pyrolysis temperature of Mg/biochar and biochar induced a decrease in yield, an increase in carbon (C) content, a decrease in hydrogen (H) content, and an increase in BET surface area. However, nitrogen (N) content did not change significantly. Moreover, Mg/biochar contained numerous magnesium nanoparticles. Both Mg/biochar and biochar were rich in organic functional groups, including O–H, C=O, C=C, and C–Cl. 

Similarly, the yield analysis (Table 2) show that yields of biochar and Mg/biochar decreased when pyrolysis temperature increased from room temperature to 300 °C. Furthermore, the weights of biochar and Mg/biochar decreased by 48.5% and 46.4%, respectively. These weights were reduced further by 16.6% and 16.7% when the pyrolysis temperature increased from 300 °C to 450 °C, respectively. The weights decreased by another 3.0% and 2.2% when the pyrolysis temperature increased from 450 °C to 600 °C, respectively. The decreasing yield trend indicated the loss of chemical composition in the corn. Raw corn generally contained roughly 12.5% lignin, 40% cellulose, and 20% hemicelluloses [22]. However, these chemical compositions were destroyed and volatilized by the increased pyrolysis temperature [23]. Moreover, the yield of Mg/biochar over than that of biochar by 2.0% to 2.8% suggested that this portion of the yield corresponded to magnesium content. 

**Table 2:** Characteristic of corn, biochar, and Mg/biochar.

Sample	Yield (%)	C (%)	H (%)	N (%)	BET-N_2_ Surface Area (m^2^/g)
corn	--	35.46	6.327	0.72	--
B300	51.5	48.82	5.642	0.70	388.193
B450	34.9	65.96	3.670	0.76	425.477
B600	31.9	70.64	2.544	0.73	494.929
Mg/B300	53.6	46.89	5.420	0.67	382.114
Mg/B450	36.9	62.33	3.470	0.72	421.492
Mg/B600	34.7	65.06	2.430	0.67	490.294

The CHN element analysis (Table 2) shows an increase in pyrolysis temperature induces an increase in C content, and a decrease in H content, although N content remains unchanged. C content was mainly related to pyrolysis temperature; therefore, it increased with this temperature. The reduced H content indicates that the H–O bond was broken and volatilized during the pyrolysis process and that additional aromatic rings were generated [24]. N content did not change significantly because the N functional groups could not form vaporized molecules. However, they generated new substances with complex structures [25]. The C, H, and N contents of Mg/biochar were lower than those of biochar because of the magnesium content in Mg/biochar. 

The BET surface area analysis (Table 2) demonstrates that the BET surface areas of Mg/biochar and biochar increased with pyrolysis temperature. It indicated the carbon structure of Mg/biochar and biochar changed according to different pyrolysis processes. When the pyrogenation temperature was lower than 500 °C, numerous mesopores were generated. When pyrogenation temperature was higher than 600 °C, mesopore structures collapsed [26]. Furthermore, the surface area of Mg/biochar was close to that of biochar at a constant pyrogenation temperature. Therefore, the embedded magnesium nanoparticles did not block the mesoporous structures. 

**Figure 1:**
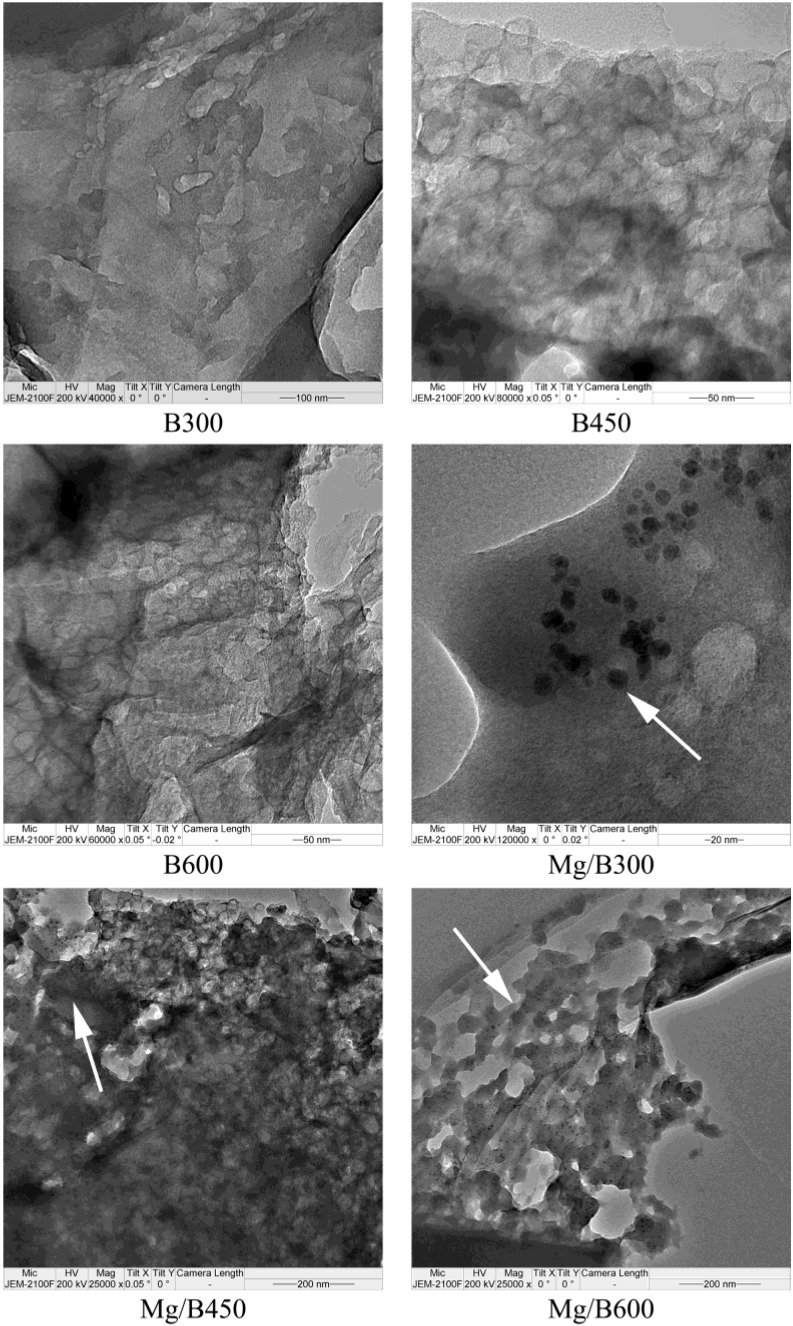
TEM analysis of biochar and Mg/biochar.

The TEM analysis (Figure 1) shows that the number of mesoporous structures increased with the increase in pyrolysis temperature. In the process, the distribution of these structures was changed from order to disorder. Lots of magnesium nanoparticles were presence in Mg/biochar. The mesoporous structure was not obvious in B300 and Mg/B300, thereby suggesting that the temperature did not reach the threshold at which mesoporous structures open. The mesoporous structures of B450 and Mg/B450 were clear and densely arranged. Portions of the mesoporous structures of B600 and Mg/B600 collapsed and aggravated the chaotic distribution of mesopores. 

**Figure 2:**
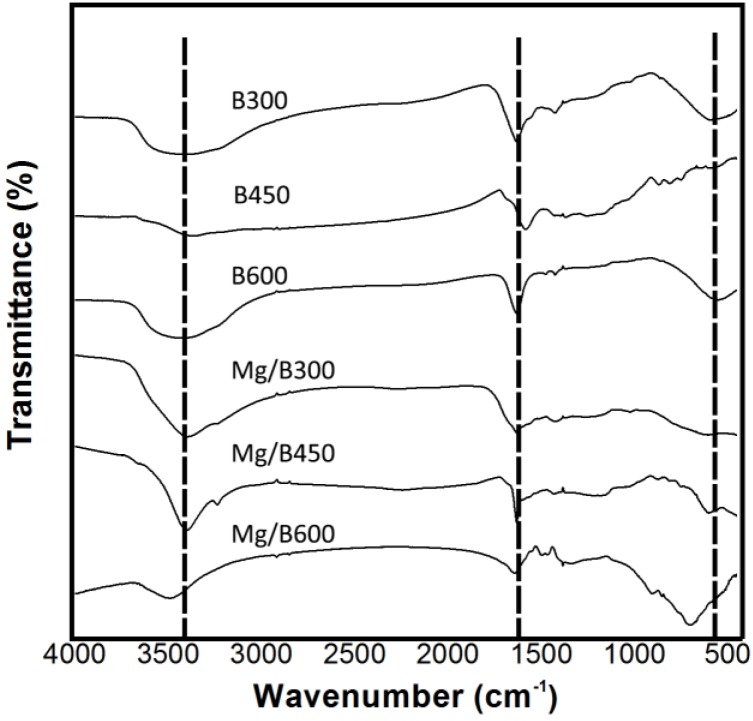
FTIR analysis of Mg/biochar and biochar.

The FTIR analysis (Figure 2) demonstrates that the character peaks of Mg/biochar are similar to those of biochar and that magnesium nanoparticles do not affect the structural formation of organic functional groups in Mg/biochar. The character peaks at 3200 cm^−1^–3700 cm^−1^, 1400 cm^−1^–1690 cm^−1^, and 550 cm^−1^–850 cm^−1^ represented the hydroxyl (O–H), double bonds (C=O and C=C), and organic chloride (C–Cl). Thus, Mg/biochar and biochar are rich in organic functional groups, such as O–H, C=O, C=C, and C–Cl, which benefit adsorption [17]. 

The surface area, number and distribution of mesopores, and organic functional groups are important factors in physical adsorption [27]. An increase in pyrolysis temperature induces increases in surface area and in the number of mesopores. Furthermore, mesopore distribution becomes disordered and organic functional groups are produced. As a result, the P adsorption capabilities of Mg/biochar and biochar are enhanced. With magnesium nanoparticles impregnated, Mg/biochar adsorption is driven by physical and chemical action, whereas biochar adsorption relies only on physical action. 

### 3.2. P Adsorption Efficiency 

#### 3.2.1. Adsorption Kinetics 

**Figure 3:**
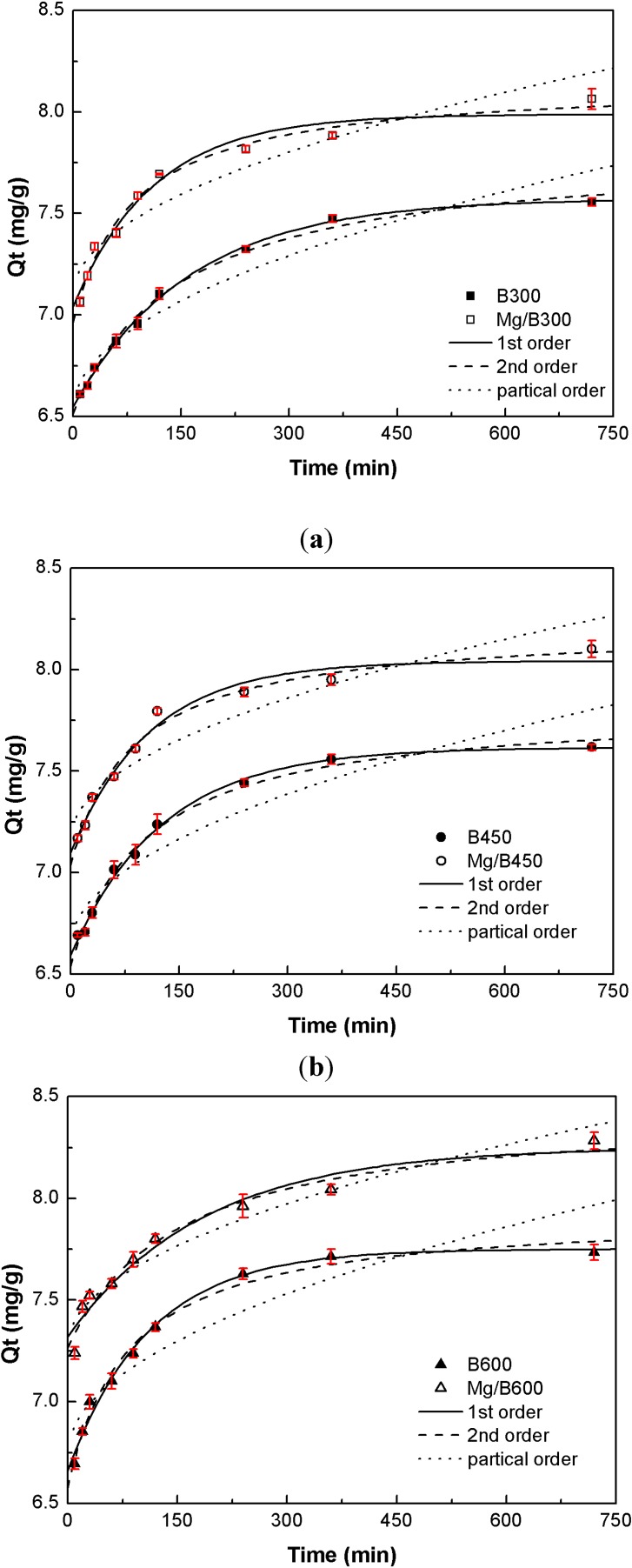
(**a**) P adsorption kinetics of Mg/B300 and B300; (**b**) P adsorption kinetics of Mg/B450 and B450; (**c**) P adsorption kinetics of Mg/B600 and B600.

The P adsorption kinetics of Mg/biochar and biochar were fitted to three kinetic models (Figure 3). The results demonstrated that the P adsorption process of Mg/biochar was mainly controlled by chemical action, whereas that of biochar was mainly driven by physical action. Furthermore, embedded magnesium nanoparticles could accelerate Mg/biochar adsorption. 

The linear equation of pseudo first-order can be expressed as follows:

ln(*Q_e_* – *Q_t_*) = ln*Q_e_* – *k*_1_*t* (1)

The linear equation of pseudo second-order can be expressed as follows:

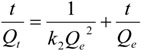
(2)


The particle diffusion equation can be expressed as follows:

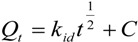
(3)
where *Q_e_* (mg·g^−1^) is the adsorbed amount of P at equilibrium, *Q_t_* (mg·g^−1^) is the adsorbed amount of P at time* t*, *k*_1_ (h^−1^), *k*_2_ (g·mg^−1^·h^−1^), and *k_id_* (g·mg^−1^·h^−1^) are reaction rate constants, and *C* is a constant related to boundary layer thickness. Table 3 shows the parameters of the three kinetic models. 

**Table 3:** Phosphorus (P) adsorption kinetics parameter of Mg/biochar and biochar.

Sample	Parameters	1st-Order	2nd-Order	Piratical-Order
B300	Parameter 1	*k*_1_ = (6.16 ± 0.36) × 10^−3^	*k*_2_ = (0.850 ± 0.122) × 10^−3^	*K_id_* = (44.1 ± 4.5) × 10^−3^
	Parameter 2	*Q_e_* = 7.57 ± 0.02	*Q_e_* = 7.81 ± 0.06	*C* = 6.53 ± 0.06
	*R*^2^	0.997	0.992	0.923
B450	Parameter 1	*k*_1_ = (7.88 ± 0.61) × 10^−3^	*k*_2_ = (1.20 ± 0.20) × 10^−3^	*K_id_* = (43.7 ± 5.7) × 10^−3^
	Parameter 2	*Q_e_* = 7.62 ± 0.02	*Q_e_* = 7.82 ± 0.05	*C* = 6.63 ± 0.08
	*R*^2^	0.994	0.989	0.880
B600	Parameter 1	*k*_1_ = (9.06 ± 1.04) × 10^−3^	*k*_2_ = (1.55 ± 0.33) × 10^−3^	*K_id_* = (45.5 ± 6.9) × 10^−3^
	Parameter 2	*Q_e_* = 7.74 ± 0.03	*Q_e_* = 7.92 ± 0.06	*C* = 6.74 ± 0.09
	*R*^2^	0.987	0.982	0.840
Mg/B300	Parameter 1	*k*_1_ = (8.90 ± 1.73) × 10^−3^	*k*_2_ = (1.45 ± 0.32) × 10^−3^	*K_id_* = (41.0 ± 5.2) × 10^−3^
	Parameter 2	*Q_e_* = 7.99 ± 0.05	*Q_e_* = 8.15 ± 0.06	*C* = 7.09 ± 0.07
	*R*^2^	0.962	0.981	0.884
Mg/B450	Parameter 1	*k*_1_ = (9.13 ± 1.47) × 10^−3^	*k*_2_ = (1.40 ± 0.30) × 10^−3^	*K_id_* = (40.4 ± 5.5) × 10^−3^
	Parameter 2	*Q_e_* = 8.04 ± 0.04	*Q_e_* = 8.21 ± 0.06	*C* = 7.16 ± 0.07
	*R*^2^	0.974	0.983	0.871
Mg/B600	Parameter 1	*k*_1_ = (5.39 ± 1.42) × 10^−3^	*k*_2_ = (0.778 ± 0.251) × 10^−3^	*K_id_* = (40.6 ± 3.7) × 10^−3^
	Parameter 2	*Q_e_* = 8.25 ± 0.09	*Q_e_* = 8.44 ± 0.11	*C* = 7.27 ± 0.05
	*R*^2^	0.944	0.962	0.938

The P adsorption process of Mg/biochar conformed to the pseudo second-order kinetic model and can adsorb 90% of the equilibrium amount of P within 30 min, thereby confirming that the P adsorption process was mainly controlled by chemical action [13]. The surface charge of biochar is generally lower, which limits its effect on P adsorption [23]. Magnesium nanoparticles can increase this surface charge, thus improving and accelerating P adsorption. The chemical electrostatic reaction of magnesium particles with P may thus be crucial to rate control. The value of* k*_2_ represents the power of P adsorption, and it decreases with an increase in pyrolysis temperature. Therefore, the P adsorption rate of Mg/biochar decelerates with the increase in pyrolysis temperature while the effect of physical adsorption increases. In other words, additional P adsorption sites are provided to reduce the effect of chemical adsorption, comparatively. 

The P adsorption process of biochar conformed to the pseudo first-order kinetic model and can adsorb up to 90% of the equilibrium amount of P after 30 min, thereby confirming that the P adsorption process was mainly driven by physical action [13]. The hydrogen bonding interaction, supply more adsorption sites, is the main reason of physical action for P adsorption by biochar. The rate constant *k*_1_ increased with the increase in pyrolysis temperature. Thus, physical adsorption was enhanced, the number of P adsorption sites increased, and the P adsorption rate of biochar accelerated. 

The particle diffusion fitting models of Mg/biochar and biochar did not pass through the origin point, thus suggesting that the P adsorption process of Mg/biochar and biochar were also influenced by the particle internal diffusion [28]. 

#### 3.2.2. Adsorption Isotherms

**Figure 4:**
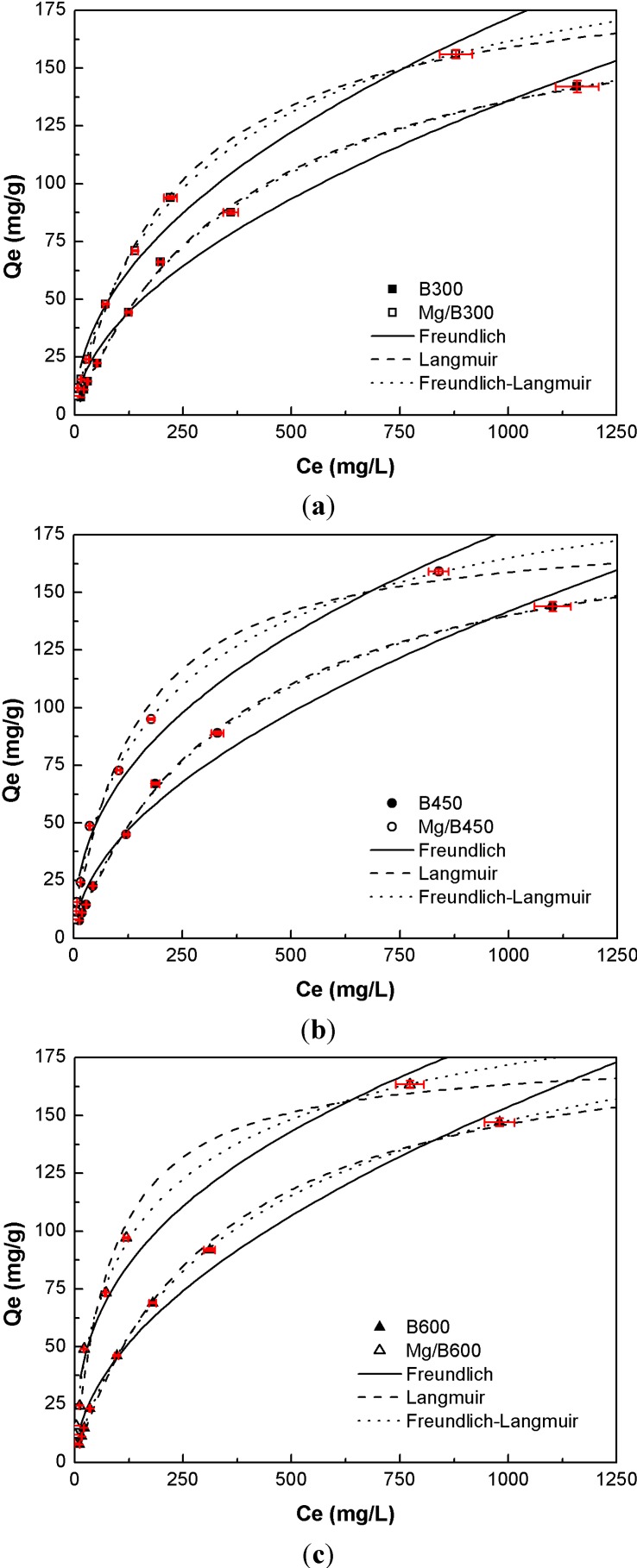
(**a**) P adsorption isotherm of Mg/B300 and B300; (**b**) P adsorption isotherm of Mg/B450 and B450; (**c**) P adsorption isotherm of Mg/B600 and B600.

The P adsorption isotherms of Mg/biochar and biochar were fitted to three adsorption isothermal models (Figure 4). Among these models, the Langmuir-Freundlich model was the one that best fit the P adsorption of both Mg/biochar and biochar. The maximum amounts of adsorbed P were 239 mg/g and 225 mg/g for Mg/biochar and bicohar, respectively. The maximum P adsorption amount of Mg/biochar was higher than that of biochar. 

The Freundlich isothermal equation for adsorption describes non-ideal adsorption on a non-uniform surface and is expressed as follows:

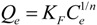
(4)


The Langmuir isothermal equation for adsorption describes the monolayer adsorption on a uniform surface and is expressed as follows:

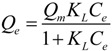
(5)


The Langmuir-Freundlich isothermal equation for adsorption integrates the empirical isothermal equations of Freundlich and Langmuir, which can be expressed as follows:

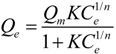
(6)
where *K_F_* [(L/g)^n^], *K_L_* (L/mg), and *K* (L/mg) are the constant of Freundlich, Langmuir, and Langmuir-Freundlich adsorption isothermal equation, respectively. A parameter relevant to the reaction strength between adsorbed molecules and adsorbent surface is 1/*n*. *Q_m_* (mg/g) denotes monolayer adsorption capacity. 

Table 4 indicates that the three models fit the P adsorption isotherms of Mg/biochar and biochar well (*R*^2^ > 0.97). Among the three models, however, the Langmuir-Freundlich model was the best fit. This finding suggests that the adsorption capabilities of P from swine wastewater by Mg/biochar and biochar were both controlled by multiple processes. The results obtained in the current study were also similar to those of previous research [11]. The value of 1/*n* represents the heterogeneity of the site energies and has been divided to five levels [29]. When the value of 1/*n* is between 1 and 0.5, the adsorption is categorized as pseudo-linear level. The smaller the value of 1/*n* is, the more favorable the adsorption is. Moreover, the 1/*n* of Mg/biochar and biochar decreased as the synthesis temperature increased. Thus, the adsorption capabilities of both Mg/biochar and biochar tended to be favorable adsorption with increased synthesis temperature. The 1/*n* of Mg/biochar was greater than that of biochar at the same synthesis temperature. Therefore, Mg/biochar can absorb P more easily than biochar, even at a low concentration level [29]. The *K* values of Mg/biochar and biochar increased with synthesis temperature increased, that suggested that the adsorption capacity increased [30]. The *K* value of Mg/biochar was higher than that of biochar at the same synthesis temperature, which indicated the adsorption capacity of Mg/biochar was larger than that of biochar at the same synthesis temperature. The maximum P adsorption amounts were 232 mg/g, 233 mg/g, and 239 mg/g for Mg/B300, Mg/B450, and Mg/B600, respectively, as fitted by the Langmiur-Freundlich model. The maximum P adsorption amounts were 200 mg/g, 201 mg/g, and 225 mg/g for B300, B450, and B600, respectively, as fitted by the Langmiur-Freundlich model. The maximum amount of P adsorbed by Mg/biochar was higher than that adsorbed by biochar, thereby demonstrating that Mg/biochar can enhance the amount of P adsorbed from swine wastewater. 

**Table 4:** P adsorption isotherm parameter of Mg/biochar and biochar.

Models		B300	B450	B600	Mg/B300	Mg/B450	Mg/B600
Freundlich	*K_F_*	3.29 ± 0.87	3.55 ± 0.94	4.05 ± 0.90	5.95 ± 1.22	9.12 ± 1.76	14.3 ± 2.4
	*n*	0.539 ± 0.041	0.534 ± 0.042	0.526 ± 0.035	0.487 ± 0.033	0.429 ± 0.032	0.371 ± 0.028
	R^2^	0.974	0.973	0.984	0.980	0.977	0.973
Langmuir	*K_L_* (×10^−3^)	2.53 ± 0.13	2.72 ± 0.14	3.15 ± 0.22	4.34 ± 0.37	7.35 ± 1.17	11.7 ± 2.3
	*Q_m_*	190 ± 4	191 ± 4	193 ± 6	196 ± 7	180 ± 11	177 ± 12
	R^2^	0.999	0.999	0.997	0.995	0.984	0.973
Langmuir-Freundlich	*K* (×10^−3^)	3.07 ± 0.50	3.30 ± 0.54	5.03 ± 0.32	7.73 ± 0.88	16.6 ± 2.1	31.0 ± 5.0
	*Q_m_*	200 ± 11	201 ± 11	225 ± 7	232 ± 14	233 ± 21	239 ± 35
	*n*	0.947 ± 0.044	0.947 ± 0.045	0.860 ± 0.020	0.823 ± 0.038	0.721 ± 0.051	0.638 ± 0.076
	R^2^	0.999	0.999	1.000	0.999	0.997	0.992

#### 3.2.3. Thermodynamic Calculation

The P adsorption capabilities of Mg/biochar and biochar were thermodynamically calculated at 288 K, 303 K, and 318 K. The Langmuir-Freundlich model was used to compute the differential enthalpy of adsorption (*∆H*), adsorption free energy (*∆G*), and adsorption entropy (*∆S*). The analysis indicated that the P adsorpiton processes of Mg/biochar and biochar were endothermic, spontaneous, and disorder increase process. 

The adsorption enthalpy of the adsorbent is closely related to the adsorption amount. When the adsorption amount is initialized at one value, the corresponding adsorption enthalpy is known as the differential enthalpy of adsorption. Its calculation formula can be expressed as follows:

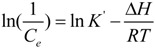
(7)
where *R* (8.314 J·mol^−1^∙K^−1^) is the ideal gas constant; *T* (K) is thermodynamic temperature; and *K*' represents a constant. Adsorption free energy can be computed using Gibbs equation, which can be expressed as follows:

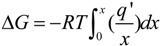
(8)
where *x* is the mole fraction of the solute in the solution; and *q*' (mmol/g) is the adsorption amount of the adsorbent. When ∆*G* is irrelevant to *q*', the formula is altered as follows:

Δ*G* = −*nRT* (9)

Adsorption entropy can be calculated using *∆H* and *∆G* with the formula below:

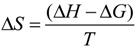
(10)


The thermodynamic parameters were given in the Table 5. The *∆H* of both Mg/biochar and biochar were positive values, thus, indicating that their P adsorptions were endothermic reaction processes. The *∆G* of both Mg/biochar and biochar were less than 0, thereby suggesting that P was spontaneous and moved from the solution to the surface of either the Mg/biochar or the biochar. In addition, the *∆G* values of Mg/biochar and biochar decreased as solution temperature increased. This result illustrated that both Mg/biochar and biochar could adsorb P efficiency at a high solution temperature [31]. The *∆S* of Mg/biochar and biochar were positive, thus, indicating that the solid-liquid interface was increasingly disordered during the P adsorption processes. Adsorption and desorption generally occurs simultaneously, and the *∆S* value increase or decrease has been depend on the effect of desorption process and the adsorption process. *∆S* value decreased with the increased in solution temperature during P adsorption by Mg/biochar and biochar, thus, illustrating that the *∆S* decreased of adsorption process became much more powerful than the *∆S* increased of desorption process at a higher solution temperature. Previous studies obtained similar results during P adsorption on ZnCl_2_ activated coir pith carbon [32] and Mg–Al layered double hydroxide [33].

**Table 5:** Phosphorus (P) adsorption thermodynamics calculation of Mg/biochar and biochar.

Sample	*T*	*∆H*	*∆G*	*∆S*
B300	288	10.8	−2.26	45.4
	303		−2.39	43.6
	318		−2.43	41.6
B450	288	10.3	−2.30	43.7
	303		−2.39	41.8
	318		−2.48	40.1
B600	288	9.87	−1.95	41.0
	303		−2.17	39.7
	318		−2.18	37.9
Mg/B300	288	10.7	−1.82	43.4
	303		−2.07	42.1
	318		−2.15	40.3
Mg/B450	288	13.5	−1.61	52.3
	303		−1.82	50.4
	318		−1.92	48.3
Mg/B600	288	20.3	−1.53	75.7
	303		−1.61	72.2
	318		−1.72	69.2

### 3.3. P Adsorption Selectivity

#### 3.3.1. Influence of pH

**Figure 5:**
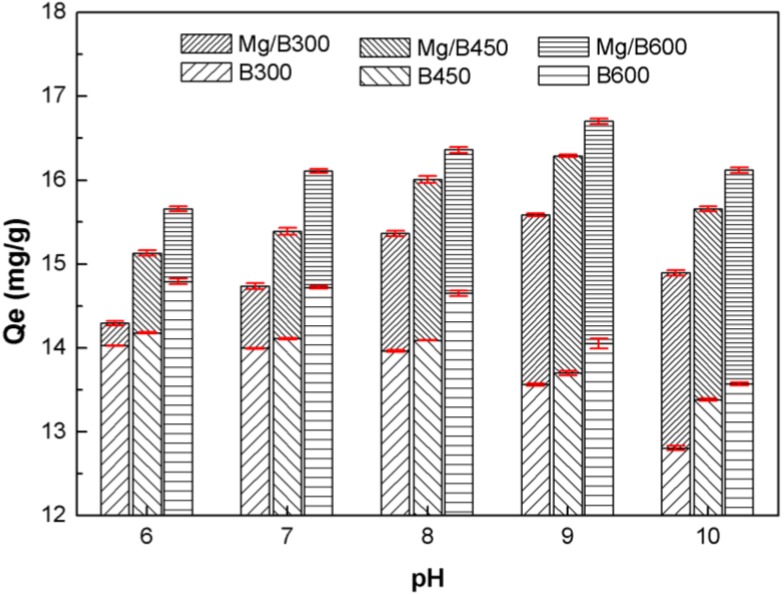
The pH influence on P adsorption of Mg/biochar and biochar.

The pH influence analysis (Figure 5) demonstrated that the resistance of Mg/biochar to pH was stronger than that of biochar because of the embedded Mg nanoparticles. As pH increased from 6 to 10, the amount of P adsorbed by Mg/biochar increased and reached its maximum amount at pH 9 before decreasing, whereas the amount of P adsorbed by biochar was decreased slightly. 

P is ternary acid whose ionization constants are 2.15, 7.20, and 12.33. When the pH is between 6 and 7.21, H_2_PO_4_^−^ is the superior in solution form. The adsorption capability of biochar relied only on physical action and was related to the quantity and distribution of mesoporous structures, surface area, and organic functional groups. However, the adsorption capability of Mg/biochar depended on a combination of physical and chemical adsorption. The chemical action of the embedded magnesium nanoparticles on P increased as pH increased. However, an acid solution environment (pH < 6) is disadvantageous for an Mg-P reaction. When pH is between 7.21 and 9, HPO_4_^2−^ is the superior in solution form. The adsorption ability of Mg/biochar increased significantly as chemical action increased. However, the adsorption capability of biochar may decline further when additional adsorption sites are consumed as a result of polynuclear interactions [13]. Similar results were obtained with carbon-based adsorbents in studies of pH effect on P recovery [34]. Nonetheless, OH^−^ competes with PO_4_^3−^ for the consumption of adsorption sites as pH increases from 9 to 10. As a result, the P adsorption amounts of Mg/biochar and biochar decreased further. 

In conclusion, Mg/biochar shows affinity toward to HPO_4_^2−^, whereas biochar shows affinity toward H_2_PO_4_^−^. The P adsorption properties of Mg/biochar are altered by the MgO nanoparticles. The optimal pH range of Mg/biochar for the selective adsorption of P is close to the pH range of swine wastewater, thus indicating that P is effectively adsorbed by Mg/biochar under the typical pH level of swine wastewater. 

#### 3.3.2. Influence of Coexisting Ions

The influence of coexisting ions on P adsorption is an important indicator of the selective P adsorption by Mg/biochar, which is stronger than that of biochar when coexisting ions are present. 

**Figure 6:**
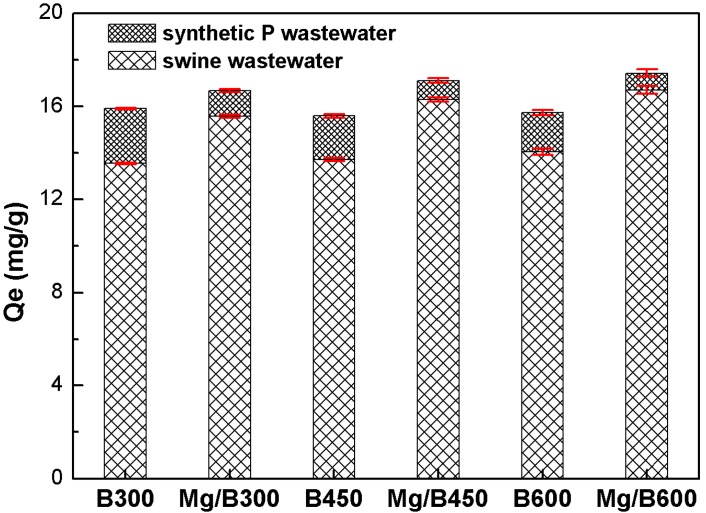
Coexisting ion influence on P adsorption of Mg/biochar and biochar.

Figure 6 shows that the amounts of P adsorbed by Mg/biochar and biochar were higher in synthetic P wastewater than that in swine wastewater. The presence of coexisting ions in swine wastewater can, thus, reduce the selective adsorption of P. The amounts of P adsorbed by Mg/B300, Mg/B450, and Mg/B600 were lower in swine wastewater than in synthetic P wastewater by 6.6%, 4.8%, and 4.2%, respectively. The amounts of P adsorbed by B300, B450, and B600 were lower in swine wastewater than in synthetic P wastewater by 14.8%, 12.1%, and 10.7%, respectively. 

The negative interaction effects for P adsorption in swine wastewater may have a twofold origin. The first is the competitive effect of coexisting anions, including Cl^−^, NO_3_^−^, and so on. Single coexisting ions, such as NO_3_^−^ (0.01M) and HCO_3_^−^ (0.01 M), could reduce the P removal ratio through biochar adsorption by 11.7% and 41.4%, respectively [13,21]. The mixture of competing compounds can also limit the amount of P adsorbed by biochar by approximately 60% [21]. The second is the co-precipitation effect of coexisting ions on the surface of Mg/biochar and biochar, such as struvite, CaHPO_4_, MgHCO_3_, and so on, which occupy adsorption sites and block the mesopores. The dominant factor in the adsorption process should be identified in future studies. Nonetheless, the results obtained in the present study were better than other results [13,21]. It indicated that the chemical action of the magnesium nanoparticles in Mg/biochar could enhance the selective adsorption of P and limit the negative interaction effects of the coexisting ions in swine wastewater. Liu* et al.* [35] applied cation-loaded material, hydroxyl–iron–lanthanum doped activated carbon fiber, to adsorb P and hindered the negative effects on P adsorption. These results were close to those of the present studies. 

Moreover, the influence of coexisting ions on P adsorption decreased with the increase in the pyrogenation temperatures of Mg/biochar and biochar. As a result, physical action was enhanced and resistance to the influence of coexisting ions was strengthened. 

### 3.4. P Available Characterization

**Figure 7:**
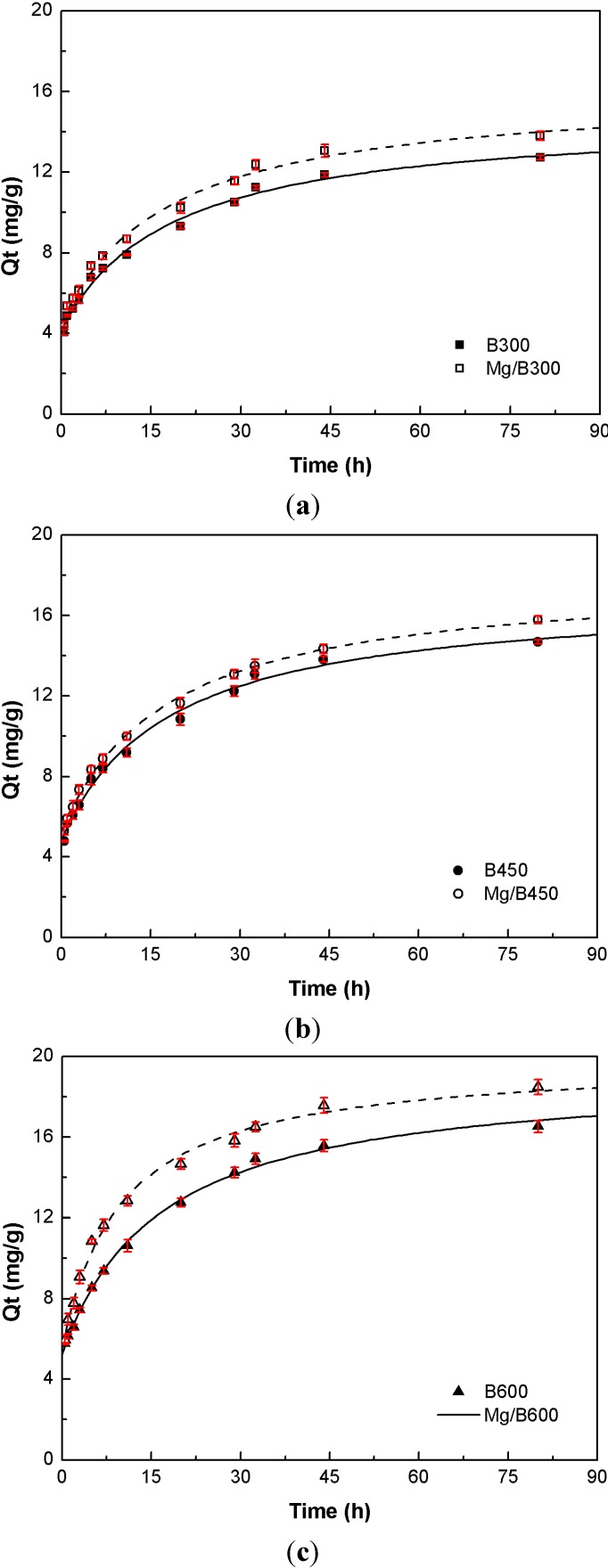
(**a**) continuous extraction of postsorption Mg/B300 and postsorption B300; (**b**) continuous extraction of postsorption Mg/B450 and postsorption B450; (**c**) continuous extraction of postsorption Mg/B600 and postsorption B600.

**Figure 8:**
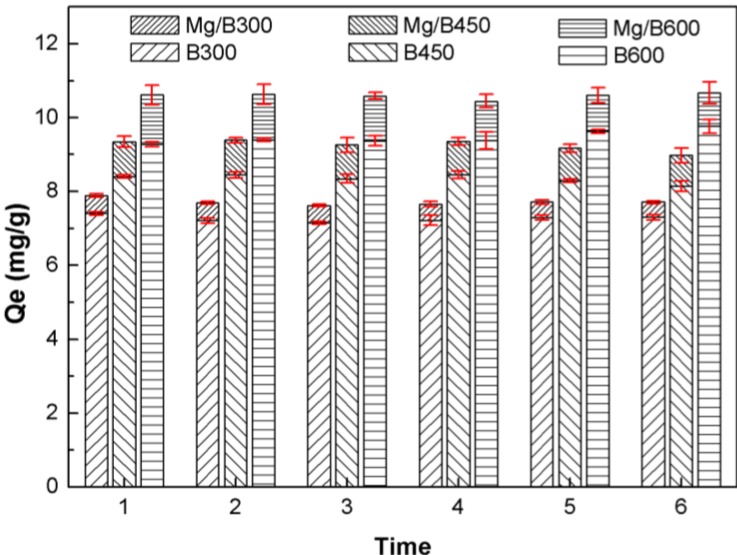
Interval extraction of postsorption Mg/biochar and postsorption biochar.

Reuse P from postsorption Mg/biochar and postsorption biochar as soil nutrient is an effective method for P recycling, because it can enhance soil fertility for plant growth. Therefore, the P release character was performed to determine the available P obtained from postsorption Mg/biochar and postsorption biochar. The continuous extraction experiments (Figure 7) were conducted to identify the P desorption kinetics characteristic of postsorption Mg/biochar and postsorption biochar. The results indicated that magnesium nanoparticles embedded in Mg/biochar do not affect P release in acid soil solution. The interval extraction experiments (Figure 8), which could simulate the situation of farm irrigation and rainwater rinse, revealed the P release character with repetitious elution. The results indicated postsorption Mg/biochar could persistently release P as with postsorption biochar. 

Figure 7 indicates that postsorption Mg/biochar, as with postsorption biochar, could gradually release P in citric acid DI. Thus, the embedded magnesium nanoparticles could not affect the P release of postsorption Mg/biochar in acid soil solution significantly. The pseudo second-order kinetic equation was used to describe the release process of P into extraction solution by postsorption Mg/biochar and postsorption biochar as follows:

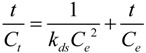
(11)
where *C_t_* (mg/L) represents P concentration at *t*; *C_e_* (mg/L) denotes P concentration at release equilibrium; and, *k_ds_* (L·mg^−1^·h^−1^) indicates the kinetic constant of second-order equation. The P release processes of Mg/biochar and biochar fit the pseudo second-order kinetic model well. Moreover, the P release equilibrium concentrations were ordered as Mg/B600 > Mg/B450 > Mg/B300 and B600 > B450 > B300, respectively. This sequence can be attributed to the total amounts of adsorbed P, which are ordered as Mg/B600 > Mg/B450 > Mg/B300 and B600 > B450 > B300. 

The interval extraction experiment result (Figure 8) suggests that postsorption Mg/biochar, as same as postsorption biochar, could persistently release P when the fresh citric acid DI was replaced, although the amount of P released was slight when the extraction solution reached equilibrium in the continuous extraction experiment. Mg/B300, Mg/B450, and Mg/B600 could release 3.3%, 3.9%, and 4.4% of total adsorbed P per time interval, whereas B300, B450, and B600 could release 3.6%, 4.1%, and 4.2% of total adsorbed P per time interval. This result confirms that postsorption Mg/biochar could persistently release P gradually for use as slow-release fertilizer. The results obtained in our research are similar to others reported results [21]. 

## 4. Conclusions 

The Mg/biochar adsorption process recovered P from swine wastewater was more effectively than biochar. The following conclusions can, thus, be obtained.

Numerous magnesium nanoparticles were found in Mg/biochar. Moreover, an increase in the pyrolysis temperatures of Mg/biochar and biochar induced a decrease in yield, an increase in C content, a decrease in H content, and an increase in BET surface area. N content does not change significantly. In addition, Mg/biochar and biochar were both rich in organic functional groups, such as O–H, C=O, C=C, and C–Cl. 

The P adsorption process of Mg/biochar was mainly controlled by chemical action, whereas that of biochar was mainly driven by physical action. Mg/biochar could adsorb 90% of the equilibrium amount of P within 30 min. Furthermore, the Langmuir-Freundlich model fitted the P adsorption isotherm of Mg/biochar and biochar best. Moreover, Mg/biochar adsorbed more P from swine wastewater with the maximum P adsorption amount of 239 mg/g, while that of biochar was 225 mg/g. The P adsorption processes of Mg/biochar and biochar were endothermic, spontaneous, and disorderly increase process. The thermodynamics calculation formulas of P adsorption processes were given as ∆H > 0, ∆G < 0, and ∆S > 0. 

The resistance of Mg/biochar to pH was stronger than that of biochar. Moreover, P adsorption capability of Mg/biochar reached its maximum at pH 9. The selective adsorption of P by Mg/biochar was stronger than that by biochar given coexisting ions. The amount of P adsorbed by Mg/B300, Mg/B450, and Mg/B600 from swine wastewater were lower than those obtained from synthetic P wastewater by 6.6%, 4.8%, and 4.2%.

The Mg nanoparticles in Mg/biochar did not affect P release significantly. Postsorption Mg/biochar persistently released P, as with postsorption biochar. The P release equilibrium concentrations were ordered as Mg/B600 > Mg/B450 > Mg/B300. Mg/B300, Mg/B450, and Mg/B600 could release 3.3%, 3.9%, and 4.4% of total adsorbed P per time interval.

Although the Mg nanoparticles embedded in biochar may increase the cost of swine wastewater treatment, this synthetic Mg/biochar is advantageous because of its increased P recovery efficiency and strong P selectivity. The synergistic effect of P recycling is significant for environmental, economic, and social development; therefore, Mg/biochar can be appropriately used to recycle P from swine wastewater.
